# SplitWise regression for capturing nonlinear effects in interpretable model selection

**DOI:** 10.1038/s41598-025-26597-7

**Published:** 2025-11-27

**Authors:** Marcell T. Kurbucz, Nikolaos Tzivanakis, Nilufer Sari Aslam, Adam M. Sykulski

**Affiliations:** 1 Institute for Global Prosperity, The Bartlett, University College London, 9-11 Endsleigh Gardens, London, WC1H 0EH UK; 2 Department of Mathematics, Faculty of Natural Sciences, Imperial College London, 180 Queen’s Gate, London, SW7 2AZ UK

**Keywords:** Stepwise regression, Interpretable modeling, Dummy variables, Threshold effects, Model selection, Software, Applied mathematics, Computational science, Software, Statistics

## Abstract

Capturing nonlinear relationships while maintaining interpretability remains a persistent challenge in regression modeling. We introduce SplitWise, a stepwise regression framework that adaptively transforms numeric predictors into threshold-based binary features using shallow decision trees—only when such transformations improve model fit according to the Akaike or Bayesian Information Criterion. This design preserves the transparency of linear models while flexibly capturing threshold-based nonlinear effects, positioning SplitWise between classical linear and interpretable nonlinear regression. SplitWise retains a single, globally linear equation that selectively incorporates data-driven thresholds—yielding models that remain straightforward to interpret and verify. Across synthetic scenarios with nonlinear signal patterns, SplitWise reduced median RMSE by 7–14% relative to the best-performing interpretable linear baseline and improved variable-selection accuracy (median Matthews Correlation Coefficient up to $\sim$0.79 vs. $\sim$0.51 for LASSO). On real datasets, SplitWise matched or slightly improved RMSE while selecting fewer predictors. For instance, on Wine Quality (White), it improved RMSE from 0.756 to 0.752 and on Wine Quality (Red) from 0.654 to 0.649, using 6–10 predictors. On Bodyfat, it achieved 3.48–3.49 RMSE with four predictors, comparable to Elastic Net (3.41–3.48 RMSE) but with smaller models.

## Introduction

Model transparency is not merely desirable—it is frequently essential for stakeholder trust, model validation, and regulatory compliance^1,2^. These demands have led to increasing interest in modeling approaches that preserve such clarity while offering greater flexibility and predictive power^3^. Statistical models that balance predictive accuracy with interpretability are highly valued across diverse domains^4,5^, including healthcare^6^, finance^7^, criminal justice^8^, environmental science^9,10^, and autonomous systems^11^. Linear regression exemplifies this principle: under standard assumptions, each coefficient can be directly interpreted as the marginal effect of a predictor on the outcome, providing a level of explainability that aligns with the expectations of regulated settings^12^. However, its simplicity entails important limitations—most notably, its reliance on linearity assumptions, which restrict its ability to model nonlinear or threshold-based effects unless these are explicitly introduced via feature engineering. In contrast, methods such as decision trees and neural networks can automatically learn complex, nonlinear relationships from data, but often at the expense of model transparency and interpretability^13,14^.

Stepwise regression^15^ is a widely used technique for variable selection in linear models. It systematically explores combinations of candidate predictors, adding or removing variables based on their statistical contribution to the model^16,17^. The method is commonly implemented through three main strategies: forward selection, which starts from an empty model and adds variables incrementally based on statistical significance; backward elimination, which begins with all candidate variables and removes the least significant ones; and bidirectional selection, which allows both addition and removal of variables at each step. Variable importance is typically assessed using F-statistics derived from t-tests on coefficient estimates, with the procedure terminating once predefined statistical criteria are met^18^. While traditional approaches have drawn criticism for overfitting and issues with statistical validity^19^, modern implementations increasingly employ selection metrics such as Akaike’s Information Criterion (AIC)^20^ and the Bayesian Information Criterion (BIC)^21,22^, which explicitly balance model fit and complexity. Despite this shift, some contemporary stepwise procedures continue to rely on fixed p-value thresholds for variable entry or removal^23^.

While stepwise methods provide a classical foundation for variable selection, regularization-based approaches have gained widespread adoption in linear modeling. Lasso regression^24^ encourages sparsity through an $\ell _1$ penalty, while ridge regression^25^ applies an $\ell _2$ penalty to shrink coefficients and mitigate multicollinearity without enforcing sparsity. Extensions such as adaptive lasso^26^ and the smoothly clipped absolute deviation (SCAD) penalty^27^ possess oracle properties, meaning they achieve consistent variable selection along with asymptotically optimal estimation. The elastic net^28^, which combines $\ell _1$ and $\ell _2$ penalties, is particularly effective when predictors are highly correlated. Further developments, such as group lasso^29^ and fused lasso^30^, extend these ideas to structured sparsity settings. Best subset selection^31^ offers exact solutions by evaluating all possible predictor combinations, though it is computationally feasible only in low-dimensional problems. These techniques have also been extended to generalized linear models and beyond^32^. To flexibly model nonlinear relationships while retaining sparsity, sparse additive models (SpAM)^33^ use penalized splines with sparsity-inducing penalties to capture smooth effects. However, most of these methods still assume linearity or smoothness, and therefore lack the ability to automatically detect discrete thresholds or breakpoints in continuous predictors.

While regularization helps mitigate overfitting, it does not resolve the structural rigidity of linear models, leaving them poorly suited to capture nonlinear or threshold-like patterns in data. Relationships between predictors and outcomes often change abruptly—for example, health risks that rise sharply beyond a certain age or blood pressure level. Decision tree algorithms^34,35^ tackle this by recursively partitioning the predictor space, producing piecewise constant or piecewise linear fits, as in the M5 model tree^36^. Recent variants improve scalability and regularization, such as PILOT^37^, which combines greedy splitting with ridge-regularized fits, and MOTR-BART^38^, which embeds linear components into Bayesian additive trees. In applied settings, hybrid approaches like logistic model trees have also proved useful^39^. However, despite their flexibility, these models often become structurally complex—relying on multiple nested splits or ensembles that obscure global relationships. Their interpretability is therefore local and path-dependent rather than global and equation-based, limiting their transparency in high-stakes or policy contexts.

Beyond tree-based methods, rule-based hybrids such as RuleFit^40^ attempt to combine predictive strength with interpretability by constructing sparse linear models from binary decision rules. These human-readable rules retain partial interpretability^41^, but the two-stage procedure—first generating large ensembles of candidate rules, then regularizing them—introduces another layer of complexity. When many overlapping rules remain, it becomes difficult to summarize their joint influence, and the resulting model no longer resembles a compact linear equation. Moreover, such methods rarely aim to systematically identify or optimize threshold-based transformations of original features with the explicit goal of improving linear model performance. This highlights a persistent methodological gap: current methods rarely introduce nonlinearity in a targeted, transparent way while maintaining the structure of a single global linear regression.

To address this limitation, we present SplitWise regression, a deterministic framework that combines adaptive threshold-based transformations with stepwise selection—a flexible, criterion-driven search that naturally accommodates both linear and thresholded predictors. Our goal is to bridge the divide between the interpretability of linear models and the flexibility of nonlinear approaches that often sacrifice transparency. The method allows numeric variables to enter the model either as standard linear terms or as binary indicators based on data-driven cut-points. Each candidate transformation is evaluated using AIC or BIC to ensure that only interpretable, predictive modifications are retained. The resulting model remains a global linear equation, augmented by a small number of thresholded features—e.g., $I(18< \text {age} < 34)$ or $I(\text {blood pressure} > 140)$—which preserve transparency while capturing relevant nonlinearity. In this sense, SplitWise occupies the middle ground between stepwise regression and more flexible rule-based or tree-based methods: it retains a single, auditable linear form while selectively incorporating threshold effects. Compared to full tree-based methods, this approach yields a compact and interpretable model that can be readily understood, audited, or used manually. To support adoption and reproducibility, we also release a user-friendly R package, SplitWise (*version 1.0.2*), which is freely available on CRAN^42^ and GitHub^43^.

The remainder of this paper is structured as follows. Section 2 introduces the SplitWise algorithm, its software implementation, and the evaluation datasets. Section 3 presents the experimental results along with illustrative interpretation examples. Finally, Section 4 concludes the paper and outlines directions for future research.

## Methods

This section presents the SplitWise algorithm, detailing its two transformation modes, then outlines the software implementation, datasets, and experimental setup.

### SplitWise regression algorithm

SplitWise is a model selection procedure that constructs interpretable regression models by not only selecting which variables to include, but also determining the most appropriate representation of each predictor. Unlike standard stepwise regression, which typically assumes fixed variable forms (e.g., linear), SplitWise simultaneously evaluates whether each predictor should enter the model as a linear term or as one or more segmented (dummy-coded) variables based on automatically determined threshold splits. This approach balances interpretability and flexibility, enabling the discovery of nonlinear, piecewise effects while preserving a simple, additive linear structure. To guard against overfitting, segmented (dummy-coded) representations are only selected when they provide a clear improvement in model fit and maintain sufficiently balanced data partitions.

**Fig. 1:**
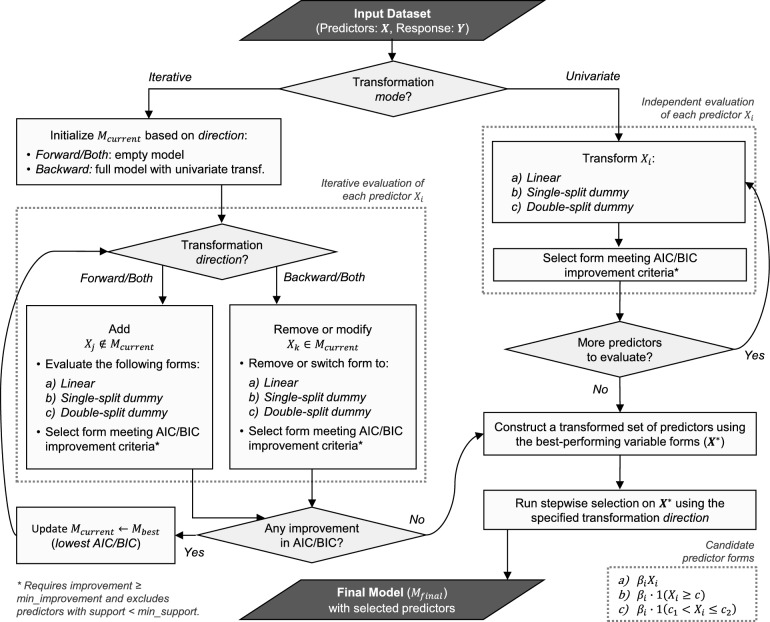
Workflow diagram of the SplitWise regression algorithm, showing the two transformation modes (iterative vs. univariate) and the sequence of steps involved in each. Unlike conventional stepwise regression, SplitWise evaluates and selects not only which variables to include, but also how each should be transformed (linear or segmented) during the modeling process. To avoid overfitting, dummy transformations are only accepted when they produce a sufficient improvement in model fit and preserve reasonably sized data partitions (denoted by min_improvement and min_support, respectively).

The algorithm supports two transformation modes—iterative and univariate—which differ in how variable transformations are evaluated and selected. These modes are illustrated in Fig. 1, which outlines the sequence of operations involved.

#### Iterative mode

In the iterative mode, SplitWise performs a dynamic, model-aware search to identify both the inclusion and the most suitable form of each predictor. This search proceeds in a stepwise fashion, where the current model is updated at each iteration by evaluating potential changes. Depending on the chosen direction—forward, backward, or bidirectional—the process begins with either an empty model or a full linear model; thereafter, variables may be reintroduced or re-encoded (e.g., from linear to thresholded form) as the search proceeds. At each step, SplitWise evaluates candidate actions—including the introduction of new variables, removal of existing ones, or modification of their representation. Each candidate is evaluated according to its AIC or BIC value, and an update is applied only when it offers a sufficient improvement in model fit while preserving adequately sized data partitions.

Importantly, this mode does not treat transformation as a one-time pre-processing step, but instead allows variable representations to evolve alongside the model. Variables may be reintroduced or re-encoded at later stages if their contribution becomes significant in the context of the current model. This iterative process continues until no further action—whether adding, removing, or transforming a variable—leads to an improvement in the selected model criterion. Upon convergence, a final global stepwise selection is applied to the transformed candidate set to construct the final model.

At each iteration, for every numeric predictor not yet dummy-encoded, we estimate candidate cut points using a shallow CART (Classification And Regression Tree) model fitted to that predictor’s partial residuals with respect to the current model. We consider at most a single binary split and a two-cutpoint split (yielding three segments). For each candidate encoding, the model is refitted and AIC/BIC values are computed; a transformation is accepted only if it improves the criterion by at least min_improvement and all induced segments satisfy the min_support constraint. Accepted encodings are added, rejected ones are discarded, and the iterative search then continues based on the updated residual structure.

#### Univariate mode

The univariate mode is designed for scalability in high-dimensional settings. Here, each predictor is evaluated independently to determine its best-fitting transformation based on its individual contribution to the response. Specifically, SplitWise considers several representations for each variable: a linear term, a single-split dummy (binary threshold), and a double-split dummy (two cut points yielding three segments). Candidate cut points for these dummy encodings are determined using shallow, pruned decision trees (maximum depth two) fitted to the relationship between the predictor and the response. The trees are used solely to identify informative threshold values—ensuring deterministic, reproducible splits that respect the min_support constraint and avoid over-partitioning. All trees are fitted with cost–complexity pruning and a fixed random seed, and candidate splits that would induce any segment below $\texttt {min\_support} \cdot n$ are discarded to ensure determinism and reproducibility.

For each predictor, model fit is evaluated using AIC or BIC under each candidate transformation. The representation yielding the lowest value is selected, provided it improves the criterion by a sufficient margin and produces adequately sized data partitions. Variables for which the null (intercept-only) model provides the best fit are discarded. The result is a transformed candidate set in which each variable appears in its most appropriate univariate form. To construct the final model, SplitWise applies a global stepwise selection procedure (using the specified direction) to this transformed candidate set. This ensures that although the transformations are selected independently, variable inclusion remains jointly optimized across the predictor set.

#### Model output

Conceptually, the iterative mode searches for transformations in a model-aware, joint fashion—using partial residuals to adapt splits to the evolving model—whereas the univariate mode pre-selects a single representation per predictor independently. The former is typically more computationally intensive but can capture dependencies with the current model fit. Both transformation modes ultimately produce a final regression model composed of a subset of predictors, each in its chosen representation. While the iterative mode explores transformations jointly within the evolving model, the univariate mode relies on independent evaluation followed by joint selection. In both cases, the result is a sparse, interpretable linear model that allows for piecewise or threshold effects where appropriate.

### Software implementation

The calculations were performed using the R programming environment (*version 4.5.1*)^44^. The SplitWise algorithm has been implemented in a dedicated R package, SplitWise (*version 1.0.2*), available as open-source software via CRAN^42^ and GitHub^43^.

The package supports both transformation modes (“iterative” and “univariate”) through a single entry-point function: splitwise(). Key user options include the direction of stepwise selection (“forward”, “backward”, or “both”), the selection criterion (AIC or BIC), and control over which variables are eligible for transformation. To prevent overfitting, the algorithm only permits segmented transformations when they produce a sufficiently large improvement in model fit (min_improvement) and maintain balanced data partitions (min_support).

To enhance interpretability and reproducibility, the returned model object (splitwise_lm class) includes comprehensive metadata describing the selected transformations, dummy split thresholds, and the final design matrix used for estimation. Custom print() and summary() methods provide human-readable reports that detail which variables were dummy-encoded and how. Additional methods—such as predict(), coef(), residuals(), fitted(), and model.matrix()—are implemented to support seamless integration with downstream workflows, enabling users to inspect, analyze, and reuse the fitted model with minimal overhead.

The package is designed to integrate cleanly into standard R modeling workflows, relying primarily on base functions such as lm() and step() for model estimation and selection. For threshold detection in numeric variables, it uses only a single external dependency: the rpart package (*version 4.1.21*)^45^. This minimal reliance on external libraries enhances robustness and ensures cross-platform compatibility—the SplitWise package is fully operating system-agnostic and functions identically across Windows, macOS, and Linux. Installation is handled via install.packages(“SplitWise”), and the package includes a README file, examples, and complete function documentation. The software is released under the GPL-3 license. More information, including benchmark scripts, example datasets, and reproducible experiments, is available at: https://github.com/mtkurbucz/SplitWise (retrieved: October 10, 2025).

#### Minimal code example

**Fig. 2:**
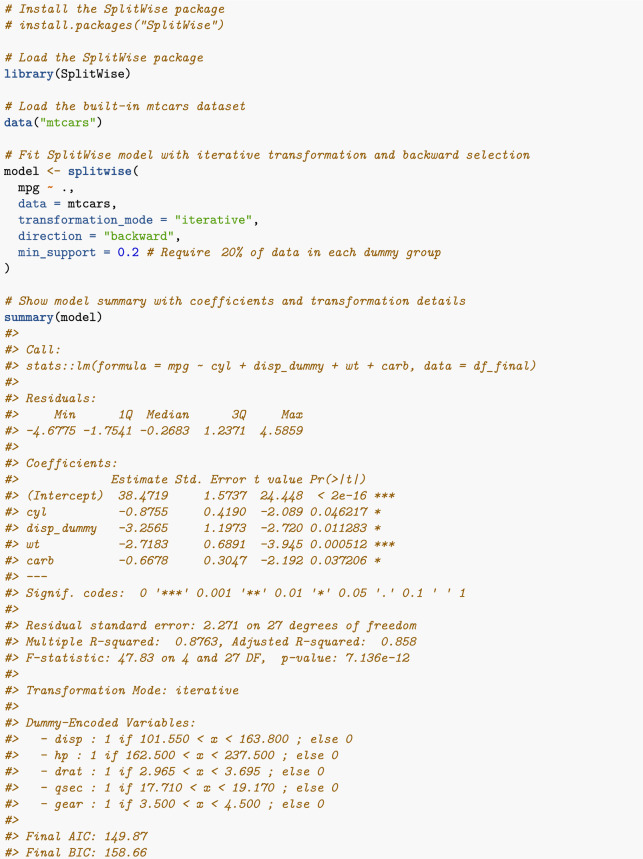
SplitWise model summary on the Mtcars dataset using iterative transformation, backward stepwise selection, and a 20% minimum group size for dummy splits.

The following minimal example demonstrates how to apply the SplitWise package to the *Mtcars* dataset^46^, which contains data on various car models, including variables such as miles per gallon (mpg), horsepower, and weight. In this example, mpg is used as the target variable, and the method combines iterative transformations with backward stepwise selection. The resulting output—shown in Figure 2—includes model coefficients, residual diagnostics, dummy-encoded variable details, and information criteria (AIC and BIC).

The final model includes an intercept and four predictors—three in linear form and one as a dummy variable—yielding a compact and interpretable outcome. Specifically, cyl, wt, and carb enter the model as significant linear terms ($\hbox {all with p-values} < 0.05$), while disp appears in a dummy-encoded form (disp_dummy) that captures whether engine displacement exceeds 101.55. This dummy variable exhibits a strong negative association with mpg, suggesting that cars with larger engines tend to have lower fuel efficiency. In contrast, several other variables—hp, drat, qsec, and gear—were considered with dummy transformations but excluded during stepwise selection. These transformation choices enable the model to flexibly capture nonlinear relationships while maintaining interpretability. Model diagnostics—including a high adjusted $R^2$ (0.858), low residual standard error (2.27), and competitive AIC and BIC values—indicate strong explanatory power without signs of overfitting.

### Datasets and experimental setup

We benchmark the SplitWise method using a combination of synthetic and real-world regression datasets, employing a consistent evaluation protocol across all experiments. Each dataset is randomly partitioned into training (70%) and test (30%) sets, with performance metrics calculated over 100 independent runs to ensure robustness against variability in data splits. Our analysis exclusively targets methods that produce interpretable models, explicitly excluding black-box algorithms that prioritize predictive accuracy at the expense of transparency.

SplitWise is compared against several established interpretable linear modeling techniques, including stepwise regression using the stats R package (*version 4.5.1*)^44^, best subset regression via the leaps package (*version 3.1*)^47^, and penalized regression methods—specifically LASSO, adaptive LASSO, and elastic net—implemented using the glmnet package (*version 4.1-10*)^32^. For penalized models, hyperparameters are tuned via cross-validation. SplitWise uses fixed parameters with min_support = 0.2. In synthetic experiments, we set min_improvement = 5 to encourage selectivity and prevent overfitting, whereas in real-world datasets, a lower threshold (min_improvement = 3) is adopted to allow greater flexibility given smaller sample sizes. These values were chosen to balance model sparsity and flexibility.

Model performance is evaluated using standard regression diagnostics, including root mean square error (RMSE), mean absolute error (MAE), computational runtime, and model complexity as measured by the number of selected predictors. For synthetic datasets with known ground truth—i.e., the set of predictors truly associated with the response—we additionally quantify variable selection accuracy using the Matthews Correlation Coefficient (MCC), defined as:

1 $$\begin{aligned} \text {MCC} = \frac{TP \times TN - FP \times FN}{\sqrt{(TP+FP)(TP+FN)(TN+FP)(TN+FN)}}, \end{aligned}$$

where *TP*, *TN*, *FP*, and *FN* respectively denote the counts of true positives (correctly selected relevant predictors), true negatives (correctly excluded irrelevant predictors), false positives (irrelevant predictors incorrectly selected), and false negatives (relevant predictors missed). Originally developed for binary classification, MCC provides a balanced measure of selection performance, particularly beneficial when the number of relevant and irrelevant predictors exhibit significant imbalance.

To provide a point of reference against interpretable nonlinear methods, we additionally include RuleFit, implemented via the pre R package (*version 1.0.8*)^48^, in the synthetic benchmark. Although RuleFit does not yield a single, globally transparent linear equation—unlike SplitWise and other interpretable linear methods—it offers a valuable comparison, as it combines decision rules with sparse linear modeling to capture nonlinear effects. Because of its hybrid structure, only RMSE was computed for comparability, since metrics such as MCC and the number of selected predictors are not directly applicable to its rule-based representation. The RuleFit model was tuned using internal cross-validation to determine the optimal regularization strength.

#### Synthetic datasets

Synthetic datasets were generated to systematically assess model performance in controlled scenarios. Each dataset contains $n = 500$ observations and $p = 20$ predictors, generated independently from a standard normal distribution, $X_j \sim \mathcal {N}(0,1)$ for $j = 1, \dots , 20$. The response variable *y* was constructed as a sum of up to three predictors:

2 $$\begin{aligned} y = \beta _1 X_1 + \beta _2 \cdot \mathbb {I}(X_2 > \tau ) + \beta _3 \cdot \left[ -8(X_3^*- 0.5)^2 + 2\right] + \varepsilon , \end{aligned}$$

where $X_3^*$ is the rescaled version of $X_3$ to the interval [0, 1], computed as $X_3^*= [X_3 - \min (X_3)]/[\max (X_3) - \min (X_3)]$, $\tau = 0.2$ is a threshold for the step function, and $\varepsilon \sim \mathcal {N}(0, \sigma ^2)$ denotes Gaussian noise. All nonzero coefficients were fixed at $\beta _1 = \beta _2 = \beta _3 = 1$ to ensure equal signal strength across active predictors. The remaining predictors, $X_4, \dots , X_{20}$, were generated as independent noise variables that do not influence the response but are included in every regression fit to evaluate each method’s ability to distinguish relevant from irrelevant predictors. The noise variance was calibrated to yield a fixed signal-to-noise ratio (SNR) of 5:

3 $$\begin{aligned} \sigma ^2 = \frac{\operatorname {Var}\left\{ \beta _1 X_1 + \beta _2 \cdot \mathbb {I}(X_2 > \tau ) + \beta _3 \cdot [-8(X_3^*- 0.5)^2 + 2]\right\} }{5}. \end{aligned}$$

Four simulation scenarios were defined by selectively activating subsets of the signal components:

A) **Linear:** $\beta _2 = 0$, $\beta _3 = 0$ (signal from $X_1$ only);
B) **Step:** $\beta _2 = 1$, $\beta _3 = 0$ (signal from $X_1$ and thresholded $X_2$);
C) **U-shape:** $\beta _2 = 0$, $\beta _3 = 1$ (signal from $X_1$ and quadratic transformation of $X_3$);
D) **Complete:** $\beta _2 = 1$, $\beta _3 = 1$ (signal includes all three components).

To reduce computational burden and keep results clear, both SplitWise and stepwise were fixed to use forward selection in all scenarios. For each scenario, the SplitWise mode (univariate or iterative) was chosen by training-set RMSE and then fixed prior to evaluating test performance; the selected configuration was applied to all simulation replications. Each scenario used 100 independently generated datasets with distinct random seeds to ensure robust estimation of performance metrics. To ensure fairness, the SplitWise mode was treated as a method-internal hyperparameter: it was selected once per scenario in a single preliminary run and then held constant across replications, analogous to standard tuning procedures; no test-set information was used.

#### Real-world datasets

To evaluate generalizability and practical utility, we applied each method to five publicly available regression datasets spanning diverse domains: health, housing, automotive engineering, and food quality. Predictor variables were analyzed on their original scales without normalization in all cases, except for adaptive LASSO, where variable-specific rescaling was performed manually by dividing each predictor by the inverse absolute value of its ordinary least squares (OLS) coefficient, as required by the method’s formulation. For consistency, internal standardization in all glmnet-based models was disabled by setting standardize = FALSE. A summary of these datasets is provided in Table 1.

**Table 1:** Summary of real-world datasets used for evaluating regression methods.

Dataset	Sample size	Predictors	Target	Source
Bodyfat	252	14	Body Fat (%)	^49^
Boston Housing	506	13	Home Value ($1000s)	^50^
Mtcars	32	10	Miles per gallon (mpg)	^46^
Wine Quality (White)	4898	11	Quality score (0–10)	^51^
Wine Quality (Red)	1599	11	Quality score (0–10)	^51^

Unlike synthetic datasets, real-world datasets are fixed and not regenerated for each replication. Instead, performance variability is estimated by repeatedly resampling each dataset into training and test partitions (70%–30%) over 100 iterations.

## Results and discussion

This section presents and discusses the empirical evaluation of regression methods on both synthetic and real-world datasets. For consistency, all reported performance metrics are summarized using the median and standard deviation, unless otherwise stated. Detailed tables with all metric values—including computational runtime and MAE, which are not shown in the main figures—are provided in the Supplementary Information.

### Synthetic datasets

**Fig. 3:**
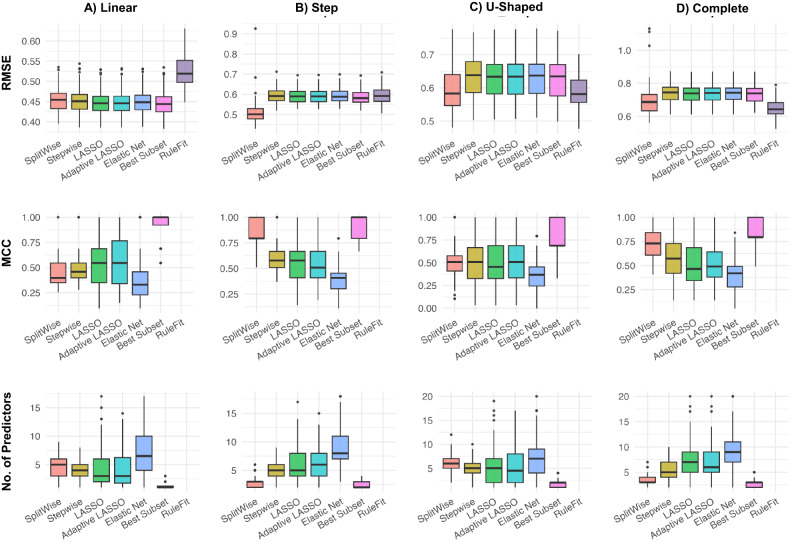
Boxplots showing RMSE, MCC, and number of predictors across four synthetic signal structures—(**A**) Linear, (**B**) Step, (**C**) U-Shaped, and (**D**) Complete. Both SplitWise and stepwise used forward selection. For SplitWise, the transformation mode (univariate or iterative) was pre-selected by lowest training-set RMSE in a single preliminary run and then held fixed across all 100 replications—a protocol analogous to hyperparameter tuning. SplitWise modes resulting from this procedure: univariate (Linear, U-Shaped), iterative (Step, Complete).

Figure 3 summarizes the performance of six variable selection methods across four synthetic scenarios, using three evaluation metrics: RMSE (lower is better), MCC (higher is better), and the number of selected predictors (lower is generally considered better for a given error). For each scenario, both SplitWise and stepwise used forward selection; the best-performing SplitWise mode (univariate or iterative, based on training-set RMSE) was selected and applied throughout all simulation replications.

SplitWise achieved the lowest RMSE among the interpretable linear models in the three nonlinear scenarios—(B) Step, (C) U-Shaped, and (D) Complete—reflecting the benefits of its adaptive dummy-encoding strategy. Wilcoxon signed-rank tests confirmed the robustness of these improvements, with $p < 0.001$ in every pairwise comparison.

Across the nonlinear scenarios, SplitWise reduced median RMSE by 7–14% relative to the next-best interpretable linear baseline (best subset), with absolute improvements of 0.052–0.079 RMSE. The largest gain occurred in the Step scenario (13.6%; 0.502 vs. 0.581), followed by U-Shaped (8.2%; 0.583 vs. 0.635) and Complete (7.0%; 0.687 vs. 0.739). As a nonlinear benchmark, RuleFit achieved slightly lower RMSE in U-Shaped (0.581 vs. 0.583) and Complete (0.642 vs. 0.687), at the cost of substantially higher runtime and reduced global interpretability, and it performed worse in the Linear and Step settings.

MAE values, as reported in the Supplementary Information, supported the same conclusion. In the (B) Step scenario, SplitWise achieved the lowest MAE at 0.400, ahead of the best subset (0.468), with other methods ranging between 0.468 and 0.474. In the (C) U-Shaped setting, SplitWise’s MAE was 0.457, compared to 0.486 for the best subset and 0.487–0.495 for the remaining methods. In the (D) Complete scenario, SplitWise achieved 0.537, again outperforming the best subset (0.579), while the other methods exceeded 0.580. These results demonstrate consistent and meaningful performance gains in nonlinear settings.

In the (A) Linear scenario, where no transformation was needed, all methods yielded nearly identical RMSE (range: 0.444–0.454) and MAE (range: 0.354–0.361). SplitWise remained competitive without overfitting or generating unnecessary dummy variables, owing to its selectivity constraints (min_support, min_improvement).

Regarding selection quality (MCC) and model sparsity (number of selected predictors), the best subset method consistently produced the most compact models and achieved the highest MCC across all scenarios. SplitWise ranked among the top methods in MCC and consistently selected the second fewest predictors, indicating a strong balance between selection accuracy and model sparsity. In contrast, the elastic net and stepwise regressions tended to select more predictors, resulting in less parsimonious models.

SplitWise’s improved performance came at a modest computational cost relative to the other interpretable methods. In the (C) U-Shaped scenario, where the univariate mode was used, the median runtime was 0.037 seconds—about 2.5 times longer than stepwise (0.014 seconds). In the (D) Complete scenario, the iterative mode was applied, yielding a median runtime of 0.176 seconds, approximately ten times that of stepwise (0.016 seconds). By contrast, the nonlinear RuleFit model required 2.6–2.7 seconds per run—nearly two orders of magnitude slower.

### Real-world datasets

**Fig. 4:**
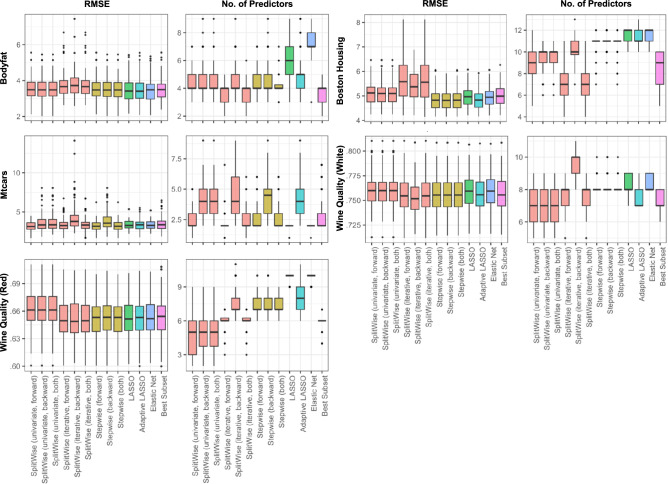
RMSE and number of selected predictors across five real-world datasets: Bodyfat, Boston Housing, Mtcars, Wine Quality (White), and Wine Quality (Red). Each boxplot summarizes performance across repeated resampling. SplitWise and stepwise methods are shown with different mode-direction configurations.

Figure 4 presents the performance of all variable selection methods across five real-world regression datasets in terms of RMSE and the number of selected predictors.

The performance of the methods on real-world datasets was nuanced, with no single model consistently dominating across all metrics. However, when model sparsity is taken into account, SplitWise achieved competitive RMSE and MAE values while maintaining substantially fewer predictors than most penalized regression methods. Overall, its strength lies in balancing predictive accuracy, interpretability, and compactness.

Across datasets, Bodyfat provides a representative example: the lowest RMSE and MAE were reached by Elastic Net (3.48 RMSE, 2.48 MAE), LASSO (3.41 RMSE, 2.52 MAE), and Adaptive LASSO (3.41 RMSE, 2.55 MAE), whereas SplitWise (univariate) remained highly competitive (3.48–3.49 RMSE, 2.64–2.67 MAE). The difference relative to Elastic Net was about 0.05 RMSE on average, but SplitWise achieved comparable performance with a smaller model (four predictors) and similar runtime (0.05–0.07 seconds). Iterative variants offered no accuracy improvement and increased runtime to 0.25–0.34 seconds.

In Mtcars, the top-performing methods were Elastic Net (3.29 RMSE, 2.79 MAE), stepwise (forward/both) (3.17 RMSE, 2.53 MAE), and SplitWise (univariate, forward) (3.14 RMSE, 2.55 MAE). The RMSE differences among them were below 0.05, but SplitWise achieved this with the same or fewer predictors (two) compared to stepwise and best subset, while penalized regressions selected up to seven. This illustrates SplitWise’s efficiency in achieving similar predictive power with a simpler model structure.

For the Boston Housing data, the lowest median RMSE and MAE were produced by Elastic Net, LASSO, Adaptive LASSO, and stepwise (around 4.82 RMSE and 3.38 MAE). SplitWise (univariate) achieved slightly higher errors (5.10–5.12 RMSE, 3.55 MAE), differing by only  0.25 RMSE from the best-performing models. Importantly, it did so with a similar or lower number of predictors (9–10 vs. 11–13) and moderate runtime (0.07–0.09 seconds). Iterative SplitWise configurations were less accurate (5.37–5.58 RMSE) and considerably slower (0.47–0.84 seconds). Relative to the best subset (4.99 RMSE, 9 predictors), the univariate SplitWise offered a balanced compromise between fit, sparsity, and stability.

The Wine Quality (White) results showed that SplitWise’s iterative mode can capture moderate nonlinearities: the iterative backward configuration achieved the lowest median RMSE (0.752) and MAE (0.586), improving RMSE by about 0.004 over the best subset (0.756). However, this came at a cost of higher computation time (0.57 seconds), while all non-iterative methods—including univariate SplitWise—finished in under 0.12 seconds. The models remained compact (8–10 predictors).

A similar trend appeared in the Wine Quality (Red) dataset. Iterative SplitWise variants again reached the best RMSE (0.649) and MAE (0.504–0.508), improving RMSE by  0.005 over the best subset (0.654). The runtime, however, was moderately higher (0.26–0.45 seconds), whereas other methods completed in under 0.06 seconds. The resulting models were highly sparse, using only six to seven predictors—roughly matching best subset and fewer than LASSO or Elastic Net, which selected up to ten.

Overall, the real-world results demonstrate that SplitWise performs competitively with established penalized and stepwise methods, offering the greatest advantages when the underlying data contain threshold-like or stepwise nonlinearities. In such cases, its adaptive transformations yield measurable accuracy improvements (typically 0.004–0.08 RMSE) while preserving a linear, interpretable structure. Conversely, in datasets characterized by smooth or globally linear relationships (e.g., Bodyfat, Boston Housing), penalized regressions such as LASSO or Elastic Net may achieve similar or slightly better fits at lower computational cost. Practitioners should therefore prefer SplitWise when interpretability and sparsity are essential, particularly for medium-sized problems with moderate nonlinear interactions that benefit from localized dummy-based encoding.

## Conclusion and future work

We introduced SplitWise, a regression methodology that addresses a persistent gap in interpretable model selection: reliably detecting nonlinear effects through adaptive, threshold-based dummy encoding while preserving a single global linear equation. Unlike standard stepwise or penalized regressions, SplitWise systematically explores both the structure and the transformation of predictors within a unified stepwise framework, allowing analysts to capture sharp cut-offs and other local nonlinearities without sacrificing global interpretability. An open-source R implementation, SplitWise, is available on CRAN ^42^ and GitHub ^43^, supporting reproducibility and further research.

Across synthetic and real-world datasets, SplitWise outperformed linear baselines (stepwise, best subset, and penalized regressions such as LASSO, adaptive LASSO, and elastic net) whenever key variables exhibited threshold-like effects, yielding higher accuracy and sparser models. In settings that were essentially linear, its performance matched that of established interpretable methods while maintaining compactness and avoiding overfitting. In terms of runtime, SplitWise was approximately 2.5$\times$ slower than stepwise in univariate mode and about 10$\times$ slower in iterative mode (e.g., $\sim$0.037 vs. 0.014 s; $\sim$0.176 vs. 0.016 s), but absolute times remained low for the dataset sizes considered. A nonlinear RuleFit benchmark sometimes achieved slightly lower RMSE on synthetic data, albeit with substantially higher runtime and reduced global interpretability; SplitWise is intended as a transparent linear alternative that still captures salient threshold effects.

Looking ahead, we see several extensions that preserve SplitWise’s core strength—global interpretability—while broadening scope: (i) a Python implementation to support wider adoption; (ii) hybrid strategies that combine SplitWise’s threshold construction with complementary selection techniques (e.g., best subset) to further enhance sparsity and fit; (iii) accelerated variants for ultra-high-dimensional problems ^52,53^; and (iv) extensions to generalized linear models (e.g., logistic regression) and time-series settings. Our guiding principle remains to expand SplitWise’s coverage of real-world problems while retaining a single, auditable linear structure augmented by a small number of data-driven thresholds.

## Supplementary Information

Supplementary Information.

## Data Availability

All datasets used in this study are publicly available from their respective sources, as cited in the manuscript. Synthetic datasets were generated using a custom data generation framework, as described in the Methods section.

## Code availability

The SplitWise R package implementing the methods described in this article is openly available at: https://github.com/mtkurbucz/SplitWise. The repository includes full documentation, reproducible examples, and benchmark scripts.

## References

[1] Caruana, R. *et al.* Intelligible models for healthcare: Predicting pneumonia risk and hospital 30-day readmission. In *Proceedings of the 21th ACM SIGKDD International Conference on Knowledge Discovery and Data Mining*, 1721–1730, 10.1145/2783258.2788613 (2015).

[2] Doshi-Velez, F. & Kim, B. Towards a rigorous science of interpretable machine learning. (2017). arXiv:1702.08608.

[3] Rudin, C. Stop explaining black box machine learning models for high stakes decisions and use interpretable models instead. *Nat. Mach. Intell.* **1**, 206–215. 10.1038/s42256-019-0048-x (2019). 35603010 10.1038/s42256-019-0048-x PMC9122117

[4] Marcinkevičs, R. & Vogt, J. E. Interpretable and explainable machine learning: A methods-centric overview with concrete examples. *Data Min. Knowl. Discov.* **13**, e1493 (2023).

[5] Kruschel, S. et al. *Challenging the performance-interpretability trade-off: An evaluation of interpretable machine learning models* (Bus. Inf. Syst. Eng. 2025).

[6] Ennab, M. & Mcheick, H. Designing an interpretability-based model to explain the artificial intelligence algorithms in healthcare. *Diagnostics* **12**, 1557 (2022). 35885463 10.3390/diagnostics12071557 PMC9319389

[7] Vuković, D. B., Dekpo-Adza, S. & Matović, S. Ai integration in financial services: A systematic review of trends and regulatory challenges. *Humanit. Soc. Sci. Commun.* **12** (2025).

[8] Berk, R., Heidari, H., Jabbari, S., Kearns, M. & Roth, A. Fairness in criminal justice risk assessments: The state of the art. *Sociol. Methods Res.* **50**, 3–44 (2021).

[9] Van Straaten, C., Whan, K., Coumou, D., Van Den Hurk, B. & Schmeits, M. Using explainable machine learning forecasts to discover subseasonal drivers of high summer temperatures in western and central europe. *Mon. Weather Rev.* **150**, 1115–1134 (2022).

[10] Park, J. et al. Interpretation of ensemble learning to predict water quality using explainable artificial intelligence. *Sci. Total Environ.* **832**, 155070 (2022). 35398119 10.1016/j.scitotenv.2022.155070

[11] Kim, J. *et al.* Toward explainable and advisable model for self-driving cars. *Appl. AI Lett.* **2** (2021).

[12] Flachaire, E., Hué, S., Laurent, S. & Hacheme, G. Interpretable machine learning using partial linear models. *Oxf. Bull. Econ. Stat.* **86**, 519–540 (2024).

[13] Breiman, L. Statistical modeling: The two cultures. *Stat. Sci.* **16**, 199–231. 10.1214/ss/1009213726 (2001).

[14] Lipton, Z. C. The mythos of model interpretability. *Commun. ACM* **61**, 36–43. 10.1145/3233231 (2018).

[15] Efroymson, M. A. Multiple regression analysis. In *Mathematical Methods for Digital Computers* (eds Ralston, A. & Wilf, H.) 191–203 (Wiley, 1960).

[16] Vàliaho, H. A synthetic approach to stepwise regression analysis. *Phys.-Math.* **34**, 91–132 (1969).

[17] Vàliaho, H. & Pekkonen, T. *A Procedure for Stepwise Regression Analysis* (De Gruyter, 1976).

[18] Miller, A., Panneerselvam, J. & Liu, L. A review of regression and classification techniques for analysis of common and rare variants and gene-environmental factors. *Neurocomputing* **489**, 466–485. 10.1016/j.neucom.2021.08.150 (2022).

[19] Harrell, F. E. *Regression Modeling Strategies* (Springer, 2001).

[20] Akaike, H. A new look at the statistical model identification. *IEEE Trans. Automatic Control* **19**, 716–723. 10.1109/TAC.1974.1100705 (1974).

[21] Schwarz, G. Estimating the dimension of a model. *Ann. Stat.* **6**, 461–464. 10.1214/aos/1176344136 (1978).

[22] Raftery, A. E. Bayesian model selection in social research. *Sociol. Methodol.* **25**, 111–163. 10.2307/271063 (1995).

[23] Fong, Y. et al. chngpt: Threshold regression model estimation and inference. *BMC Bioinform.* **18**, 454. 10.1186/s12859-017-1863-x (2017). 10.1186/s12859-017-1863-x PMC5644082 29037149

[24] Tibshirani, R. Regression shrinkage and selection via the lasso. *J. R. Stat. Soc. Series B (Stat. Methodol.)* **58**, 267–288 (1996).

[25] Hoerl, A. E. & Kennard, R. W. Ridge regression: Biased estimation for nonorthogonal problems. *Technometrics* **12**, 55–67 (1970).

[26] Zou, H. The adaptive lasso and its oracle properties. *J. Am. Stat. Assoc.* **101**, 1418–1429 (2006).

[27] Fan, J. & Li, R. Variable selection via nonconcave penalized likelihood and its oracle properties. *J. Am. Stat. Assoc.* **96**, 1348–1360 (2001).

[28] Zou, H. & Hastie, T. Regularization and variable selection via the elastic net. *J. R. Stat. Soc. Series B (Stat. Methodol.)* **67**, 301–320 (2005).

[29] Yuan, M. & Lin, Y. Model selection and estimation in regression with grouped variables. *J. R. Stat. Soc. Series B (Stat. Methodol.)* **68**, 49–67 (2006).

[30] Tibshirani, R., Saunders, M., Rosset, S., Zhu, J. & Knight, K. Sparsity and smoothness via the fused lasso. *J. R. Stat. Soc. Series B (Stat. Methodol.)* **67**, 91–108 (2005).

[31] Hastie, T., Tibshirani, R. & Friedman, J. *The Elements of Statistical Learning* 2nd edn. (Springer, 2017).

[32] Friedman, J., Hastie, T. & Tibshirani, R. Regularization paths for generalized linear models via coordinate descent. *J. Stat. Softw.* **33**, 1–22 (2010). 20808728 PMC2929880

[33] Ravikumar, P., Lafferty, J., Liu, H. & Wasserman, L. Sparse additive models. *J. R. Stat. Soc. Series B (Stat. Methodol.)* **71**, 1009–1030 (2009).

[34] Breiman, L., Friedman, J. H., Olshen, R. A. & Stone, C. J. *Classification and Regression Trees* (Wadsworth International, 1984).

[35] Quinlan, J. R. *C4.5: Programs for Machine Learning* (Morgan Kaufmann, 1993).

[36] Quinlan, J. R. Learning with continuous classes. *Proceedings of the 5th Australian Joint Conference on Artificial Intelligence* 343–348 (1992).

[37] Raymaekers, J., Rousseeuw, P. J., Verdonck, T. & Yao, R. Fast linear model trees by pilot. *Mach. Learning* **113**, 6561–6610 (2024).

[38] Prado, E. B., Moral, R. A. & Parnell, A. C. Bayesian additive regression trees with model trees. *Stat. Computing* **31**, 1–13 (2021).

[39] Adeyemo, V. E., Balogun, A. O., Mojeed, H. A., Akande, N. O. & Adewole, K. S. Ensemble-based logistic model trees for website phishing detection. In *Advances in Cyber Security: Second International Conference, ACeS 2020, Penang, Malaysia, December 8-9, 2020, Revised Selected Papers 2*, 627–641 (Springer, 2021).

[40] Friedman, J. H. & Popescu, B. E. Predictive learning via rule ensembles. *Ann. Appl. Stat.* **2**, 916–954. 10.1214/07-AOAS148 (2008).

[41] Molnar, C. *Interpretable Machine Learning* (Leanpub, 2022). https://christophm.github.io/interpretable-ml-book/.

[42] Kurbucz, M. T., Tzivanakis, N., Aslam, N. S. & Sykulski, A. M. *SplitWise: Hybrid Stepwise Regression with Single-Split Dummy Encoding*, 10.32614/CRAN.package.SplitWise (2025). R package version 1.0.2.

[43] Kurbucz, M. T., Tzivanakis, N., Aslam, N. S. & Sykulski, A. SplitWise R Package (2025). 10.1038/s41598-025-26597-7 PMC12660875 41309860

[44] R Core Team. *R: A Language and Environment for Statistical Computing*. R Foundation for Statistical Computing, Vienna, Austria (2025). Version 4.5.1.

[45] Therneau, T., Atkinson, B. & Ripley, B. *Recursive Partitioning and Regression Trees* (2025). R package version 4.1-24.

[46] Henderson, H. V. & Velleman, P. F. Building multiple regression models interactively (1981).

[47] Lumley, T. *Regression Subset Selection* (2024). R package version 3.2.

[48] Fokkema, M. Fitting prediction rule ensembles with r package pre. *J. Stat. Softw.* **92**, 1–30 (2020).

[49] Johnson, R. W. Statistical modeling of body fat percentage. *Available from StatLib Data Archive* (1996).

[50] Harrison, D. Jr. & Rubinfeld, D. L. Hedonic housing prices and the demand for clean air. *J. Environ. Econ. Manag.* **5**, 81–102 (1978).

[51] Cortez, P., Cerdeira, A., Almeida, F., Matos, T. & Reis, J. Modeling wine preferences by data mining from physicochemical properties. *Decision Support Syst.* **47**, 547–553 (2009).

[52] Stippinger, M. et al. Biometricblender: Ultra-high dimensional, multi-class synthetic data generator to imitate biometric feature space. *SoftwareX* **22**, 101366 (2023).

[53] Hanczár, G. et al. Feature space reduction method for ultrahigh-dimensional, multiclass data: Random forest-based multiround screening (rfms). *Mach. Learn. Sci. Technol.* **4**, 045012 (2023).

