# Supplementary material for: SplitWise regression for capturing nonlinear effects in interpretable model selection
Source: Sci Rep. 2025 Nov 27;15:42454. doi: 10.1038/s41598-025-26597-7 (PMC12660875; doi:10.1038/s41598-025-26597-7)
Supplement: Supplementary file 1 — Supplementary Information. [file 41598_2025_26597_MOESM1_ESM.pdf]

Supplementary Information for "SplitWise Regression for Capturing Nonlinear Effects in Interpretable Model Selection"

Marcell T. Kurbucz[1,*], **Nikolaos Tzivanakis**[1], **Nilufer Sari Aslam**[1], **and Adam M. Sykulski**[2]

[1]Institute for Global Prosperity, The Bartlett, University College London, 9-11 Endsleigh Gardens, London, WC1H 0EH, United Kingdom
[2]Department of Mathematics, Faculty of Natural Sciences, Imperial College London, 180 Queen's Gate, London, SW7 2AZ, United Kingdom
[*]m.kurbucz@ucl.ac.uk

Contents

Supplementary Table S1: Results on Synthetic Datasets 2
Supplementary Table S2: Results on Real-World Datasets 3

Supplementary Table S1: Results on Synthetic Datasets

Method	MAE_med	MAE_SD	RMSE_med	RMSE_SD	Time_med	Time_SD	NumPred_med	NumPred_SD	MCC_med	MCC_SD
**Linear**										
SplitWise (univariate, forward)	0.361	0.027	0.454	0.031	0.034	0.017	5	1.872	0.397	0.125
Stepwise (forward)	0.359	0.027	0.451	0.032	0.012	0.004	4	1.570	0.459	0.139
LASSO	0.357	0.027	0.446	0.031	0.014	0.003	3	3.503	0.546	0.280
Adaptive LASSO	0.353	0.026	0.443	0.030	0.013	0.018	1	0.946	1.000	0.094
Elastic Net	0.356	0.026	0.448	0.031	0.015	0.016	6	3.700	0.350	0.168
Best Subset	0.354	0.027	0.444	0.031	0.002	0.001	1	0.514	1.000	0.145
RuleFit	0.412	0.033	0.519	0.041	2.645	0.094	–	–	–	–
**Step**										
SplitWise (iterative, forward)	0.400	0.052	0.502	0.058	0.158	0.065	3	0.971	0.793	0.148
Stepwise (forward)	0.472	0.031	0.591	0.037	0.014	0.004	5	1.484	0.577	0.125
LASSO	0.471	0.031	0.589	0.036	0.014	0.011	5	3.239	0.577	0.211
Adaptive LASSO	0.468	0.031	0.584	0.036	0.017	0.016	4	2.855	0.667	0.255
Elastic Net	0.474	0.031	0.589	0.036	0.014	0.004	9	3.403	0.369	0.150
Best Subset	0.468	0.030	0.581	0.035	0.002	0.001	2	0.518	1.000	0.100
RuleFit	0.471	0.037	0.592	0.043	2.700	0.103	–	–	–	–
**U-Shape**										
SplitWise (univariate, forward)	0.457	0.048	0.583	0.064	0.037	0.006	6	1.775	0.509	0.160
Stepwise (forward)	0.491	0.044	0.638	0.058	0.014	0.005	5	1.810	0.509	0.211
LASSO	0.487	0.044	0.633	0.058	0.015	0.004	4	3.951	0.509	0.243
Adaptive LASSO	0.488	0.045	0.632	0.059	0.016	0.003	2	3.293	0.688	0.199
Elastic Net	0.495	0.044	0.637	0.057	0.015	0.005	7	4.012	0.369	0.198
Best Subset	0.486	0.045	0.635	0.059	0.002	0.002	2	0.756	0.688	0.192
RuleFit	0.458	0.037	0.581	0.046	2.642	0.101	–	–	–	–
**Complete**										
SplitWise (iterative, forward)	0.537	0.076	0.687	0.093	0.176	0.067	3	0.931	0.728	0.167
Stepwise (forward)	0.588	0.044	0.745	0.057	0.016	0.005	5	1.757	0.572	0.181
LASSO	0.580	0.043	0.739	0.055	0.015	0.004	7	3.715	0.490	0.204
Adaptive LASSO	0.581	0.044	0.734	0.056	0.017	0.003	4	2.808	0.728	0.219
Elastic Net	0.584	0.042	0.742	0.054	0.015	0.016	9	3.531	0.404	0.168
Best Subset	0.579	0.043	0.739	0.057	0.002	0.002	3	0.802	0.793	0.130
RuleFit	0.511	0.041	0.642	0.049	2.702	0.076	–	–	–	–

Table S1. Median (med) and standard deviation (SD) of performance metrics across synthetic datasets. MAE: mean absolute error; RMSE: root mean squared error; Time: runtime in seconds (from model fitting to evaluation); NumPred: number of selected predictors; MCC: Matthews correlation coefficient (selection accuracy). Both SplitWise and stepwise used forward selection. For SplitWise, the transformation mode (univariate or iterative) was pre-selected by lowest training-set RMSE in a single preliminary run and then held fixed across all replications—analogous to standard hyperparameter tuning.

Supplementary Table S2: Results on Real-World Datasets

Method	MAE_med	MAE_SD	RMSE_med	RMSE_SD	Time_med	Time_SD	NumPred_med	NumPred_SD
**Bodyfat**								
SplitWise (univariate, forward)	2.668	0.442	3.486	0.602	0.0532	0.0293	4	0.855
SplitWise (univariate, backward)	2.642	0.459	3.481	0.634	0.0591	0.0095	4	1.093
SplitWise (univariate, both)	2.642	0.458	3.481	0.632	0.0739	0.0104	4	1.093
SplitWise (iterative, forward)	2.798	0.483	3.661	0.700	0.2541	0.0769	4	0.797
SplitWise (iterative, backward)	2.881	0.555	3.723	0.751	0.2685	0.0832	4	1.191
SplitWise (iterative, both)	2.798	0.506	3.661	0.731	0.3368	0.1431	4	0.821
Stepwise (forward)	2.641	0.438	3.477	0.595	0.0205	0.0053	4	0.859
Stepwise (backward)	2.629	0.454	3.472	0.628	0.0159	0.0053	4	1.094
Stepwise (both)	2.658	0.440	3.476	0.596	0.0258	0.0215	4	0.716
LASSO	2.515	0.483	3.415	0.679	0.0432	0.0092	6	1.129
Adaptive LASSO	2.548	0.451	3.405	0.658	0.0600	0.0100	5	1.341
Elastic Net	2.476	0.480	3.484	0.670	0.0449	0.0087	7	0.859
Best Subset	2.657	0.423	3.495	0.615	0.0032	0.0012	4	0.572
**Boston Housing**								
SplitWise (univariate, forward)	3.550	0.209	5.122	0.432	0.0881	0.0283	9	1.846
SplitWise (univariate, backward)	3.548	0.211	5.097	0.427	0.0712	0.0230	10	1.048
SplitWise (univariate, both)	3.548	0.211	5.097	0.427	0.0826	0.0140	10	1.048
SplitWise (iterative, forward)	3.993	0.796	5.581	0.895	0.6043	0.1643	7	1.383
SplitWise (iterative, backward)	3.844	0.455	5.374	0.602	0.4709	0.1493	10	1.185
SplitWise (iterative, both)	3.993	0.800	5.560	0.898	0.8377	0.2734	7	1.422
Stepwise (forward)	3.382	0.215	4.826	0.412	0.0554	0.0122	11	0.767
Stepwise (backward)	3.380	0.210	4.817	0.400	0.0144	0.0037	11	0.438
Stepwise (both)	3.382	0.214	4.826	0.411	0.0761	0.0237	11	0.783
LASSO	3.419	0.216	4.967	0.416	0.0454	0.0220	12	0.554
Adaptive LASSO	3.366	0.211	4.832	0.404	0.0652	0.0091	11	0.570
Elastic Net	3.423	0.215	4.944	0.408	0.0481	0.0064	12	0.542
Best Subset	3.480	0.215	4.991	0.413	0.0040	0.0012	9	1.547
**Mtcars**								
SplitWise (univariate, forward)	2.554	0.512	3.144	0.596	0.0462	0.0081	2	0.907
SplitWise (univariate, backward)	2.751	0.875	3.349	1.043	0.0638	0.0265	4	1.439
SplitWise (univariate, both)	2.754	0.868	3.363	1.034	0.0802	0.0274	4	1.410
SplitWise (iterative, forward)	2.639	0.737	3.263	0.826	0.1860	0.0815	2	0.750
SplitWise (iterative, backward)	3.094	1.590	3.787	1.866	0.2493	0.0934	4	1.611
SplitWise (iterative, both)	2.665	0.800	3.332	0.871	0.2236	0.1077	2	0.871
Stepwise (forward)	2.526	0.547	3.174	0.655	0.0147	0.0057	2	1.048
Stepwise (backward)	2.806	0.924	3.571	1.176	0.0186	0.0068	4	1.616
Stepwise (both)	2.526	0.571	3.174	0.677	0.0173	0.0077	2	0.957
LASSO	2.770	0.411	3.280	0.545	0.0305	0.0082	2	0.141
Adaptive LASSO	2.650	0.630	3.364	0.751	0.0521	0.0078	4	1.627
Elastic Net	2.789	0.402	3.286	0.539	0.0348	0.0082	2	0.100
Best Subset	2.661	0.734	3.370	0.899	0.0032	0.0019	2	1.094
**Wine Quality (White)**								
SplitWise (univariate, forward)	0.591	0.012	0.760	0.017	0.1227	0.0372	7	0.876
SplitWise (univariate, backward)	0.591	0.012	0.760	0.017	0.0996	0.0214	7	0.876
SplitWise (univariate, both)	0.591	0.012	0.760	0.017	0.0921	0.0326	7	0.876
SplitWise (iterative, forward)	0.587	0.012	0.755	0.018	0.7422	0.1371	8	0.782
SplitWise (iterative, backward)	0.586	0.011	0.752	0.017	0.5693	0.1121	10	0.656
SplitWise (iterative, both)	0.587	0.012	0.755	0.018	1.0169	0.1727	8	0.772
Stepwise (forward)	0.588	0.011	0.755	0.017	0.0884	0.0415	8	0.356
Stepwise (backward)	0.588	0.011	0.755	0.017	0.0460	0.0076	8	0.403
Stepwise (both)	0.588	0.011	0.755	0.017	0.1121	0.0305	8	0.356
LASSO	0.591	0.012	0.759	0.017	0.0865	0.0581	8	0.522
Adaptive LASSO	0.588	0.011	0.756	0.017	0.0949	0.0530	7	0.549
Elastic Net	0.592	0.012	0.759	0.017	0.0866	0.0638	8	0.500
Best Subset	0.588	0.011	0.756	0.017	0.0072	0.0031	7	0.601
**Wine Quality (Red)**								
SplitWise (univariate, forward)	0.518	0.017	0.661	0.021	0.0661	0.0263	5	1.411
SplitWise (univariate, backward)	0.518	0.017	0.661	0.021	0.0641	0.0227	5	1.383
SplitWise (univariate, both)	0.518	0.017	0.661	0.021	0.0783	0.0307	5	1.383
SplitWise (iterative, forward)	0.508	0.015	0.649	0.020	0.3556	0.0546	6	0.772
SplitWise (iterative, backward)	0.504	0.015	0.649	0.020	0.2601	0.0681	7	1.041
SplitWise (iterative, both)	0.508	0.015	0.649	0.020	0.4461	0.0958	6	0.772
Stepwise (forward)	0.506	0.015	0.653	0.020	0.0496	0.0104	7	0.720
Stepwise (backward)	0.506	0.016	0.653	0.021	0.0248	0.0234	7	0.903
Stepwise (both)	0.506	0.015	0.653	0.020	0.0623	0.0304	7	0.686
LASSO	0.506	0.015	0.651	0.020	0.0604	0.0230	10	0.327
Adaptive LASSO	0.506	0.015	0.653	0.021	0.0782	0.0126	8	1.450
Elastic Net	0.506	0.015	0.652	0.020	0.0600	0.0119	10	0.273
Best Subset	0.508	0.015	0.654	0.021	0.0045	0.0027	6	0.687

Table S2. Median (med) and standard deviation (SD) of performance metrics for all methods across five real-world datasets. MAE: mean absolute error; RMSE: root mean squared error; Time: runtime in seconds, measured from model fitting to evaluation; NumPred: number of selected predictors.
